# Runt-Related Transcription Factor 3 Promotes Acute Myeloid Leukemia Progression

**DOI:** 10.3389/fonc.2021.725336

**Published:** 2021-10-12

**Authors:** Wenwen Zhang, Qian Ma, Bing Long, Zhangyi Sun, Lingling Liu, Dongjun Lin, Minyi Zhao

**Affiliations:** ^1^ Department of Hematology, The Seventh Affiliated Hospital, Sun Yat-sen University, ShenZhen, China; ^2^ Key Laboratory of Stem Cells and Tissue Engineering, Zhongshan School of Medicine, Sun Yat-sen University, Ministry of Education, Guangzhou, China; ^3^ Department of Clinical Laboratory, Peking University First Hospital, Beijing, China; ^4^ Department of Hematology, The Third Affiliated Hospital, Sun Yat-sen University, Guangzhou, China

**Keywords:** RUNX3, super-enhancer, acute myeloid leukemia, cell cycle, apoptosis, DNA repair

## Abstract

Acute myeloid leukemia (AML) is an aggressive hematological malignancy with high relapse/refractory rate. Genetic and epigenetic abnormalities are driving factors for leukemogenesis. RUNX1 and RUNX2 from the Runt-related transcription factor (RUNX) family played important roles in AML pathogenesis. However, the relationship between RUNX3 and AML remains unclear. Here, we found that *RUNX3* was a super-enhancer-associated gene and highly expressed in AML cells. The Cancer Genome Atlas (TCGA) database showed high expression of *RUNX3* correlated with poor prognosis of AML patients. We observed that *Runx3* knockdown significantly inhibited leukemia progression by inducing DNA damage to enhance apoptosis in murine AML cells. By chromatin immunoprecipitation sequencing (ChIP-seq) analysis, we discovered that RUNX3 in AML cells mainly bound more genes involved in DNA-damage repair and antiapoptosis pathways compared to that in normal bone marrow cells. *Runx3* knockdown obviously inhibited the expression of these genes in AML cells. Overall, we identified *RUNX3* as an oncogene overexpressed in AML cells, and *Runx3* knockdown suppressed AML progression by inducing DNA damage and apoptosis.

## Introduction

Acute myeloid leukemia (AML) is one of the most common hematologic malignancies that is characterized by clonal expansion of abnormally differentiated myeloid blasts (1, 2). High treatment failure rate of AML that is caused by frequent relapses and limited treatments challenges the clinical management of AML (3). Genetic and epigenetic abnormalities, such as NPM1 mutation, DNMT3a mutation, and MLL rearrangement, are determinants of AML pathogenesis and always relate to AML prognosis (4). Consequently, it is imperative to further decipher the genetic and epigenetic characteristics of AML to identify more new molecular targets for AML treatment improvement.

Super-enhancer is a special enhancer identified to enhance the transcription of key oncogenes in various cancer cells, such as prostate cancer cells, T cell acute lymphocytic leukemia cells, and multiple myeloma cells (5–7). In mouse AML cells, some genes critical in leukemogenesis, including *Myc*, *Meis1*, and *Runx2*, are also super-enhancer-associated genes (8), which indicates that super-enhancers may dedicate to AML pathogenesis.

The Runt-related transcription factor (RUNX) family consists of three members, RUNX1, RUNX2, and RUNX3. The tumor-related functions of RUNX1 and RUNX2 were well studied. Especially, RUNX1 plays a critical role in AML pathogenesis (9). Meanwhile, RUNX3 was the least investigated (10). As a transcription factor, the heterodimer of RUNX3 and a beta subunit form a complex that binds to the core DNA sequence found in a number of enhancers and promoters and further activate or suppress transcription (11). RUNX3 was previously regarded as a tumor-suppressor gene for its inactivation promotes the progression of gastric cancer, lung cancer, colorectal cancer, and bladder cancer by upregulating oncogenes, such as *YBX1* and *GLI1*, or abrogating ARF–P53 pathway (12–16). Conversely, RUNX3 is overexpressed and exhibits oncogenic activities in ovarian cancers, basal cell carcinomas, and head and neck cancers (17–19). As well, RUNX3 overexpression drives the transformation of myelodysplastic syndrome, another myeloid malignancy, by repressing RUNX1 (20) and predicts poor prognosis in childhood AML (21). However, the relationship between RUNX3 and AML pathogenesis remains mysterious.

In this study, we discovered that RUNX3 is a super-enhancer-associated gene and highly expressed in AML cells. *Runx3* knockdown in murine AML cells efficiently impeded AML progress. Furthermore, we proved that RUNX3 bound and upregulated the expression of genes involved in DNA repair and antiapoptosis pathways to promote AML progression.

## Results

### *RUNX3* Is a Super-Enhancer-Associated Gene Only Highly Expressed in Acute Myeloid Leukemia Cells Instead of in Normal Blood Cells

**Figure 1:**
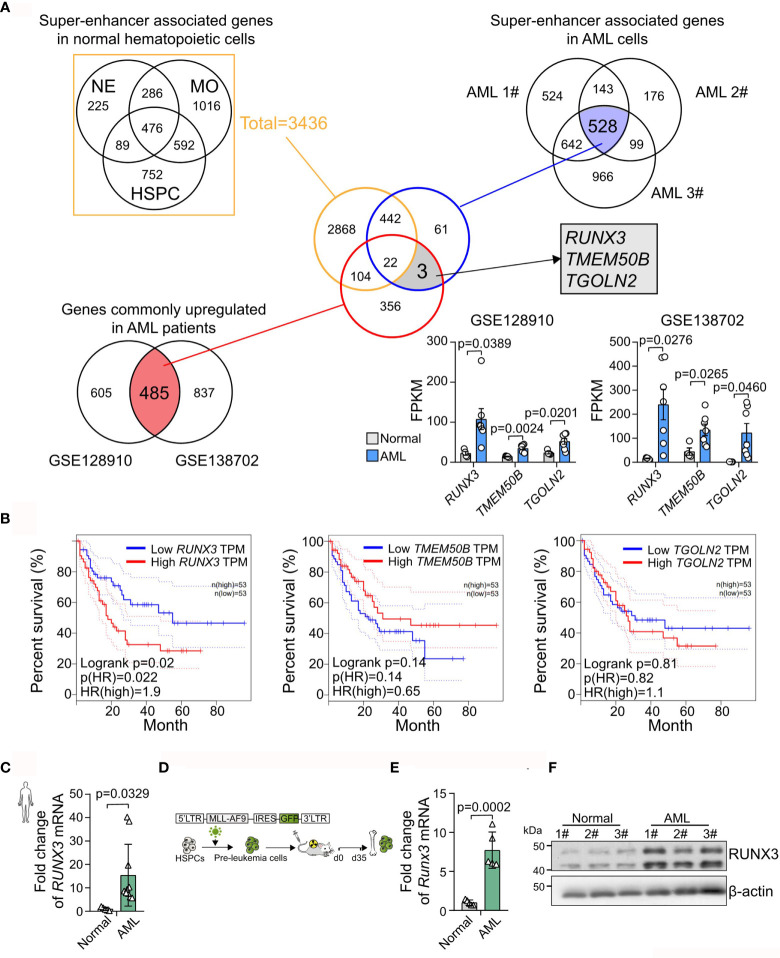
*RUNX3* is a super-enhancer-associated gene only highly expressed in acute myeloid leukemia (AML) cells instead of in normal blood cells. **(A)** Experimental scheme to search for specific and highly expressed super-enhancer-associated genes in AML cells. **(B)** The Kaplan–Meier survival curves of *RUNX3*, *TMEM50B*, and *TGOLN2* in The Cancer Genome Atlas (TCGA)-LAML database. **(C)** The *RUNX3* mRNA expression of bone marrow cells from healthy volunteers (n = 5) or AML patients (n = 10). **(D)** Experimental scheme for panels **(E, F)**. **(E)** qRT-PCR and **(F)** Western blot showing that RUNX3 expression was increased in murine leukemic cells from primary transplant mice compared with normal mouse bone marrow cells. ACTIN was used to show equal loading. Symbols represent an individual mouse.

To identify super-enhancer-associated genes that are unique in AML cells, we analyzed H3K27ac chromatin immunoprecipitation sequencing (ChIP-Seq) data of three types of normal blood cells, including neutrophils (NEs), monocytes (MOs), and hematopoietic stem cell progenitor cells (HSPCs), and AML cells (AML1#–3#). We found 3,436 super-enhancer-associated genes in normal blood cells. Meanwhile, 528 super-enhancer-associated genes were consistently identified in AML cells **(** **Figure 1A** **)**. Furthermore, two independent RNA sequencing (RNA-seq) datasets (GSE128910 and GSE138702) were analyzed and revealed that 485 genes were identified as being differentially expressed (adjusted p < 0.05) with ≥1.5-fold differential expression between the groups were consistently overexpression. Accordingly, we found three abnormally highly expressed genes (*RUNX3*, *TMEM50B*, and *TGOLN2*) those were super-enhancer-associated genes only in AML cells **(** **Figure 1A** and **Supplementary Figure S1** **)**. Furthermore, we analyzed The Cancer Genome Atlas (TCGA) database and found that only *RUNX3* expression was positively associated with poor prognosis in AML (*RUNX3*, p = 0.02; *TMEM50B*, p = 0.14; *TGOLN2*, p = 0.81; **Figure 1B**). Moreover, we observed that the expression level of *RUNX3* was remarkably higher in bone marrow cells from AML patients than that from healthy volunteers (15.39-fold increase; **Figure 1C** and **Table 1**). Consistently, both mRNA and protein levels of RUNX3 in MLL-AF9-induced murine AML cells were significantly elevated compared to those in normal murine bone marrow cells (7.72-fold increase of *Runx3* mRNA expression; **Figures 1D** **–F**).

**Table 1:** Clinical sample information.

	Sample number	Patient's identification number	Age	Subtype	Tumor burden (%) in BM	Red blood cell (RBC,10^12/L)	White blood cell (WBC,10^9/L)	Platelet (10^9/L)
**AML Patients**	1 #	695438	29	M5	29.8	66	25.9	55
	2#	665972	19	M2	36.3	63	49.9	23
	3#	687524	34	M1	75.7	39	36.6	75
	4#	742586	49	M5	54.6	38	46.4	45
	5#	675823	58	M5	79.2	48	62.1	46
	6#	668545	44	M1	68.2	56	66.6	45
	7#	698657	35	M1	58.3	44	35.5	68
	8#	699982	61	M5	59.1	55	73.2	38
	9#	659325	52	M4	27.5	67	21.1	48
	10#	695837	61	M4	52.8	50	45.5	61
	**Sample Number**	**Gender**	**Age**					
**Healthy Volunteers**	1#	Male	36					
	2#	Female	42					
	3#	Female	21					
	4#	Male	28					
	5#	Male	31					

Clinical sample information of 10 acute myeloid leukemia (AML) patients and five healthy volunteers for **Figure 1C**.

Taken together, we demonstrate that *RUNX3* is a super-enhancer-associated gene only highly expressed in AML cells instead of in normal blood cells and probably exerts pro-tumor function on AML cells.

### *Runx3* Knockdown Inhibits Acute Myeloid Leukemia Progression *In Vivo*

**Figure 2:**
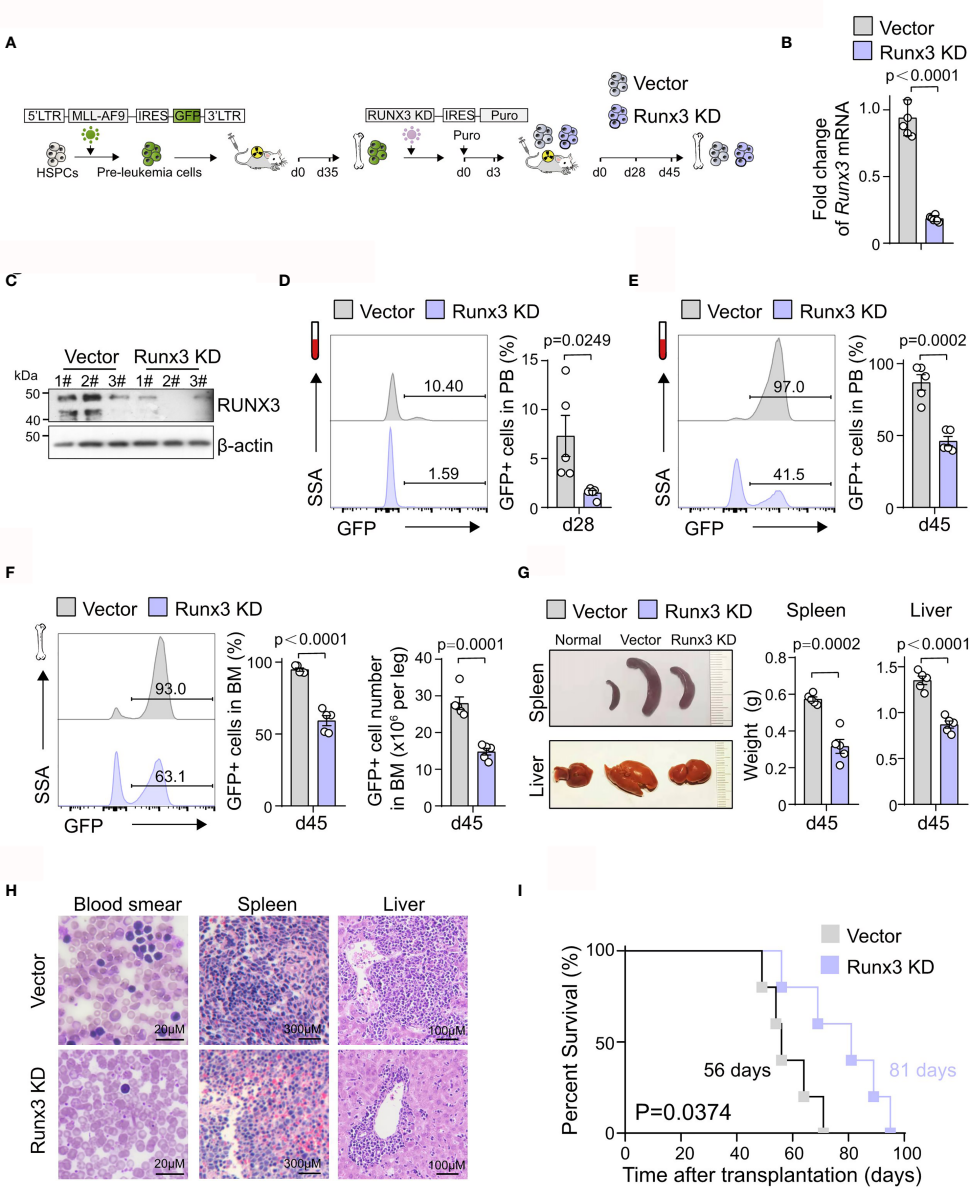
*Runx3* knockdown inhibits acute myeloid leukemia (AML) progression *in vivo*. **(A)** Experimental scheme for investigating RUNX3 role in AML progression *in vivo*. **(B)** qRT-PCR analysis showing *Runx3* knockdown in sorted AML cells from bone marrow of scramble control (Vector) and *Runx3* knockdown (*Runx3* KD) AML mice at day 45 posttransplantation. Each dot represents a mouse. **(C)** Western blot analysis showing RUNX3 knockdown. **(D)** Representative cytometric flow plots (left) and statistic results (right) show that *Runx3* knockdown decreases leukemia burden in peripheral blood (PB) at day 28 posttransplantation (n = 5 mice). **(E)** The percentage of green fluorescent protein (GFP)^+^ AML cells in the PB at day 45 posttransplantation (n = 5 mice). **(F)** Representative cytometric flow plots (left), the percentage of GFP^+^ AML cells (middle), and the number of GFP^+^ leukemic cells (right) in bone marrow (BM) at day 45 posttransplantation (n = 5 mice). **(G)** Representative image of spleen (upper left), liver (bottom left), and quantitative analysis of spleen weight (middle) and liver weight (right) from scramble control and *Runx3* knockdown AML mice (n = 5 mice). **(H)** Wright–Giemsa staining of blood smear and H&E staining of spleen and liver from scramble control and *Runx3* knockdown AML mice. Scale bar: blood smear 20 µm, spleen 300 µm, liver 100 µm. **(I)** Survival analysis of mice transplanted with scramble control or *Runx3* knockdown AML cells. Data shown are combined from two independent transplants. (n = 5 mice). p = 0.0374, log-rank test.

To further explore the potential pro-tumor role of *Runx3* in AML, equal numbers of control (Vector) or *Runx3* knockdown (*Runx3* KD) murine AML cells were transplanted into syngeneic wild-type (WT) recipients (**Figure 2A**). To evaluate the effects of *Runx3* reduction, we first sorted green fluorescent protein (GFP)^+^ leukemia cells from Vector and *Runx3* KD AML mice and confirmed that shRNA specific for *Runx3* led to decreased RUNX3 expression by qRT-PCR and Western blot analysis (80.3% reduction of *Runx3* mRNA expression; **Figures 2B** **, C** and **Supplementary Figure S2**). We found that *Runx3* KD significantly ablated AML cells in the peripheral blood (PB) [79.5% reduction on day 28 (d28), 46.9% reduction on d45; **Figures 2D** **, E**] and reduced disease burden in the bone marrow (37.4% reduction on leukemic cell frequency, 47.2% reduction on leukemic cell number; **Figure 2F**). Furthermore, the spleen and liver of AML mice were significantly enlarged, and *Runx3* knockdown significantly alleviated these symptoms (44.9% and 35.7% reduction of spleen weight and liver weight, respectively; **Figure 2G**). Consistently, histological analysis showed that AML mice in *Runx3* KD group had fewer leukemia cell infiltration in the peripheral blood, spleen, and liver **(** **Figure 2H**). Importantly, *Runx3* knockdown significantly prolonged the survival of AML mice [Median overall survival (MOS) 81 days in *Runx3* knockdown group compared to 56 days in vector control group; **Figure 2I**].

These results demonstrate that *Runx3* knockdown suppresses the progression of MLL-AF9-induced AML in mice, which supports our hypothesis that *Runx3* is oncogenic in AML.

### *Runx3* Knockdown Induces DNA Damage and Apoptosis in Acute Myeloid Leukemia Cells *In Vivo*

**Figure 3:**
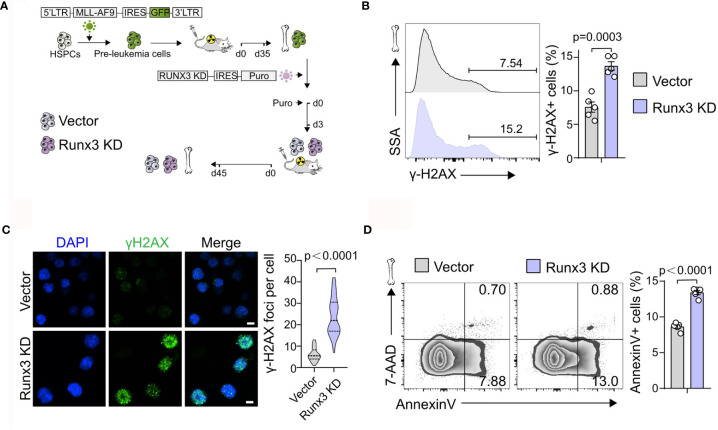
*Runx3* knockdown induces DNA damage and apoptosis in AML cells *in vivo*. **(A)** Experimental scheme for getting *Runx3* knockdown AML cells induced by MLL-AF9. **(B)** Representative flow cytometry (FCM) plots and quantitative analysis show the percentage of γ-H2AX^+^ cells in scramble control and *Runx3* knockdown AML cells (n = 5 mice). **(C)** CLSM images of γ-H2AX expression and quantification of γ-H2AX foci per cell (30 cells in each group) in scramble control and *Runx3* knockdown AML cells. Nuclear DNA was counterstained with DAPI. Scale bar = 50 μm. **(D)** *Runx3* knockdown increases apoptosis. Representative FCM plots (left) and statistical results (right) show the percentage of apoptotic cells in scramble control and *Runx3* knockdown AML cells. Bone marrow cells were stained for Annexin V and 7-AAD (n = 5 mice).

We next investigated how *Runx3* knockdown suppressed the development of AML. Through flow cytometric analysis of control (Vector) or *Runx3* KD AML cells, we found a slight increase in the percentage of G2/S/M-phase cells and G0-phase cells, accompanied by a minor decrease in the percentage of G1-phase cells after *Runx3* knockdown (1.14-fold and 1.34-fold increase in G2/S/M-phase cells and G0-phase cells, 12.8% reduction in G1-phase cells, respectively; **Figure 3A** and **Supplementary Figure S3**). Reduction of *Runx3* in leukemic cells therefore appeared to induce G0/G1 arrest, which was consistent with a reduction of leukemia burden. Furthermore, *Runx3* knockdown obviously increased DNA damage in AML cells (1.81-fold increase in γ-H2AX^+^ cells, 3.93-fold increase in γ-H2AX foci per cell; **Figures 3B, C** **)**. Annexin V and 7-AAD double staining showed that upon *Runx3* knockdown, the percentage of total apoptotic cells in AML cells was remarkably increased to 13.4% compared with 8.6% in control group **(**1.56-fold increase) (**Figure 3D**).

Altogether, these results illustrate that *Runx3* knockdown induces DNA damage and apoptosis in leukemia cells *in vivo*.

### RUNX3 Binds to Cell Cycle-Related Genes in Both Normal Bone Marrow Cells and Acute Myeloid Leukemia Cells but Specifically to DNA Repair and Antiapoptosis-Related Genes Only in Acute Myeloid Leukemia Cells

**Figure 4:**
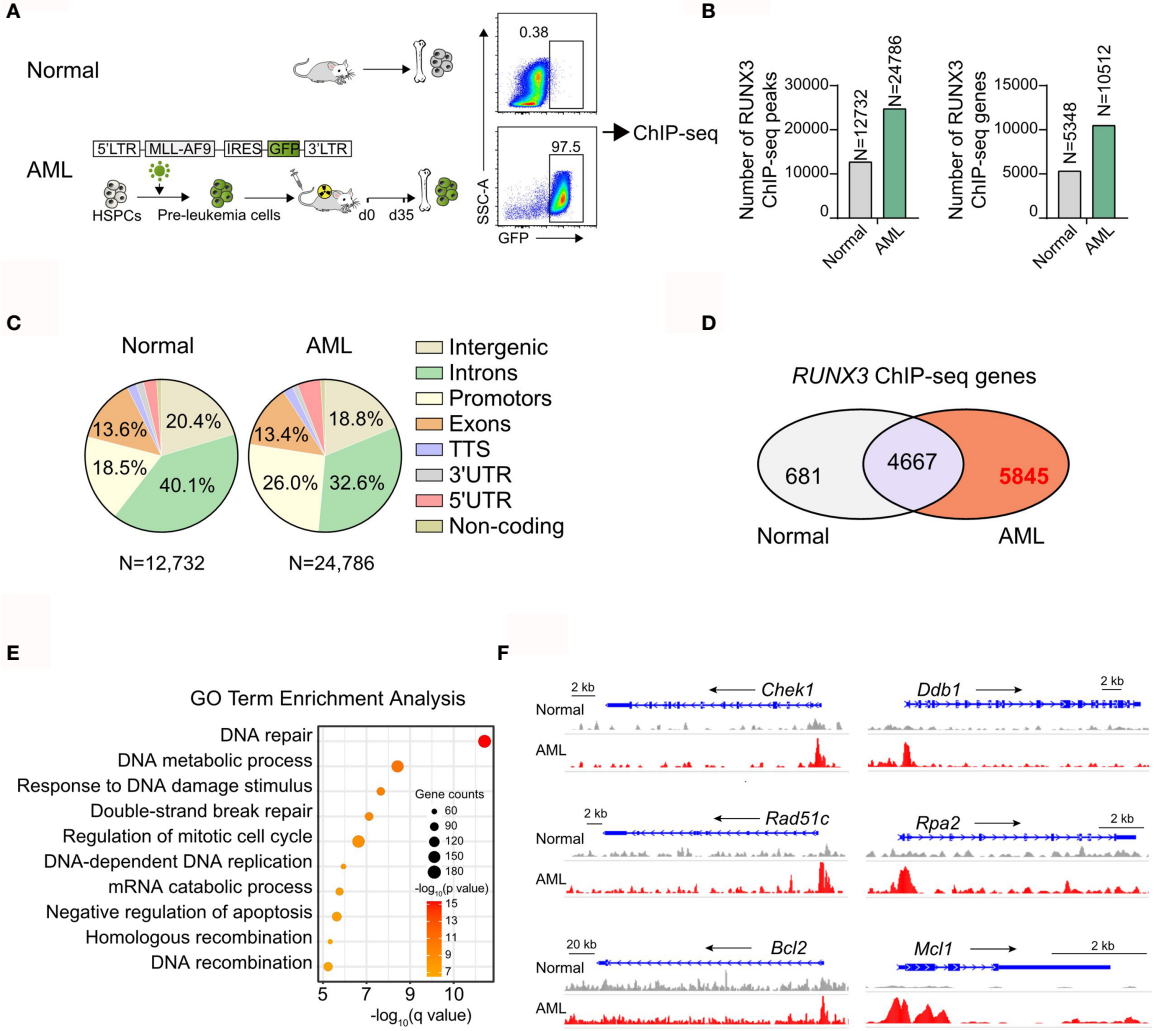
RUNX3 specifically to DNA repair and anti-apoptosis related genes only in AML cells. Chromatin immunoprecipitation sequencing (ChIP-seq) results for RUNX3 in whole bone marrow cells from normal mice or MLL-AF9 AML mice. **(A)** Experimental scheme. **(B)** Number of RUNX3 ChIP-seq peaks and genes identified by HOMER. **(C)** Pie charts show the genomic distribution of ChIP-seq peaks for RUNX3 in whole bone marrow cells from normal mice (left) or MLL-AF9 AML mice (right). Representation of the annotated regions is shown for comparison. **(D)** Venn diagram of the RUNX3-bound 5,348 genes in normal bone marrow cells and the RUNX3-bound 10,512 genes in AML bone marrow cells. **(E)** Gene Ontology (GO) term enrichment analysis of 5,845 genes that can be bound by RUNX3 in leukemia cells but not be bound in normal bone marrow cells. **(F)** Genome browser views of the distribution of RUNX3 ChIP-seq peaks in DNA repair (*Chek1*, *Ddb1*, *Rad51c*, and *Rpa2*)- and antiapoptosis (*Bcl-2* and *Mcl-1*)-related gene loci.

To determine the molecular mechanism of the oncogenic activity of RUNX3 in AML cells, we analyzed the genomic distribution of RUNX3 in bone marrow cells from normal mice and AML mice by RUNX3 chromatin immunoprecipitation followed by next-generation sequencing (ChIP-seq). The bone marrow cells from primary AML mice were collected 35 days after transplantation at which point the percentage of AML cells (GFP^+^ cells) was 97.5% **(** **Figure 4A** and **Supplementary Figures S4A, B** **)**. The number of RUNX3 peaks and corresponding genes in the leukemia group was significantly higher than those in the normal cells **(** **Figure 4B**). RUNX3 peaks were enriched at introns, promoters, intergenic sites, and exons of genes in normal cells and leukemia cells **(** **Figure 4C**). By analyzing RUNX3-bound genes in normal bone marrow cells and AML cells, we found that 4,667 genes were able to be bound by RUNX3 in both normal bone marrow cells and AML cells. There were 5,845 RUNX3-bound genes that could be found in AML cells but not in normal bone marrow cells **(** **Figure 4D**).

To further explore the difference between RUNX3-bound genes in normal bone marrow cells and AML cells, Gene Ontology (GO) enrichment analysis was performed. We found that most of the 4,667 RUNX3-bound genes in both types of cells were cell cycle-related genes, but the RUNX3 peak values were higher in AML cells **(** **Supplementary Figures S4C, D** **)**. More importantly, most of the 5,845 genes specifically bound by RUNX3 in AML cells were related to DNA repair and the negative regulation of apoptosis, such as *Chek1*, *Ddb1*, *Rad51c*, *Rpa2*, *Bcl-2*, and *Mcl-1* **(** **Figures 4E, F** **)**. Surprisingly, many classical AML-related oncogenes that have been reported were found to be bound by RUNX3 in AML cells, such as *Myc*, *Cd93* (22), *Kit*, *Ikzf2* (23), *Fto* (24), and *Sox4* (25) **(** **Supplementary Figure S4E**). Furthermore, we discovered that RUNX3 tended to bind with these classical genes related to DNA repair, antiapoptosis, and leukemogenesis around their promoter areas **(** **Figure 4F** and **Supplementary Figure S4E**). Interestingly, RUNX3 bound some classical DNA-repair genes at their enhancer areas, while it bound no antiapoptotic genes at their enhancer areas (**Supplementary Figure S4F**).

These results indicate that RUNX3 probably directly regulates genes related to cell cycle, DNA repair, and apoptosis in AML cells.

### *Runx3* Knockdown Inhibits the Expression Levels of Genes Involved in DNA Repair, Antiapoptosis, and Cell Cycle Pathways in Acute Myeloid Leukemia Cells

**Figure 5:**
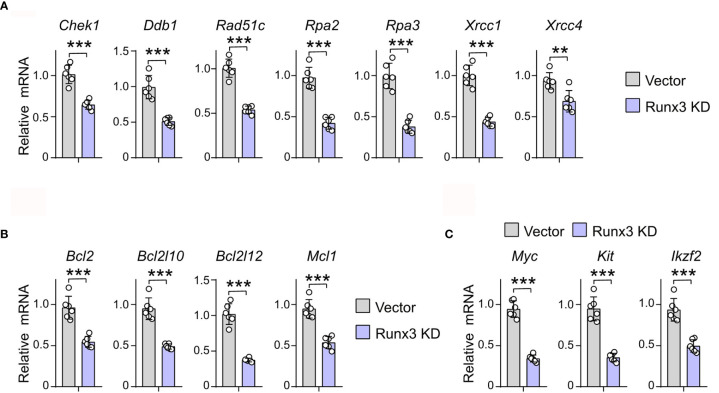
*Runx3* knockdown inhibits the expression levels of genes involved in DNA repair and anti-apoptosis pathways in AML cells. **(A–C)** The relative mRNA expression level of DNA repair **(A)**, antiapoptosis **(B)**, and leukemogenesis **(C)** related genes that RUNX3 binds to only in AML cells. Data represent mean ± SEM of six mice. **p < 0.01, ***p < 0.001. ns, not significant.

To prove the regulatory role of RUNX3 in the expression of DNA repair, antiapoptosis, and cell cycle-related genes that it binds to, we detected the expression of the above genes in murine AML cells under *Runx3* knockdown by qRT-PCR. Transcriptional analysis showed that *Runx3* knockdown decreased the expression level of cell cycle-related genes that RUNX3 binds to, such as *Cdk4*, *Ccnd1*, *Ccnd2*, *Cdk2*, *Ccna1*, and *Ccnb1* (36.4%, 21.1%, 52.3%, 52.0%, 29.9%, and 45.4% reduction, respectively) in murine AML cells (**Supplementary Figure S5**). More importantly, in murine AML cells, *Runx3* knockdown also significantly reduced the expression of DNA-repair (*Chek1*, *Ddb1*, *Rad51c*, *Rpa2*, *Rpa3*, *Xrcc1*, and *Xrcc4*) (36.4%, 48.7%, 46.4%, 56.8%, 62.1%, 56.3%, and 28.6% reduction, respectively)- and antiapoptosis (*Bcl2*, *Bcl2l10*, *Bcl2l12*, and *Mcl1*) (43.3%, 48.5%, 63.3%, and 43.4% reduction, respectively)-related genes that RUNX3 binds to only in AML cells (**Figures 5A, B**). Consistently, the expression of several genes associated with leukemogenesis that has been reported was obviously reduced after RUNX3 knockdown in murine AML cells (*Myc*, *Kit*, and *Ikzf2*) (63.8%, 62.6%, and 47.1% reduction, respectively) (**Figure 5C**).

Taken together, these results illustrate that *Runx3* knockdown inhibits the expression levels of cell cycle-, DNA repair-, antiapoptosis-, and leukemogenesis-related genes in AML cells.

## Discussion

Genetic and epigenetic abnormalities drive leukemogenesis and determine the prognosis of AML (4). They are complex and dynamically evolving (26). There is still much work to do to uncover the full genetic and epigenetic landscape of AML. In our study, we found that RUNX3 was an obvious highly expressed gene in AML cells. According to TCGA-LAML database, the high expression of RUNX3 was positively related to poor prognosis of AML patients. These discoveries suggest a potential role of RUNX3 in AML progression.

As reported, diverse solid tumors, including gastric, colorectal, lung, and bladder cancers, exhibit low expression of RUNX3 (27). Low expression of RUNX3 is caused by gene deletion and epigenetic alteration. Epigenetic alteration is the most common one (28). The *RUNX3* gene is regulated by two promoters, P1 and P2. At the P2 promoter, there is a large CpG island that is often hypermethylated in tumor cells to silence RUNX3 (29). However, the P2 promoter in AML cells is unmethylated, and demethylating agents fail to increase RUNX3 expression level in AML cells (21). Moreover, we discovered that *RUNX3* in AML cells was regulated by a super-enhancer that had strengthened transcriptional regulating ability. These evidences explain why differs from that in other solid tumor cells, RUNX3 expression in AML cells is elevated.

The function of RUNX3 in cancers is controversial. Primarily, RUNX3 was reported as a tumor-suppressor gene in multiple cancers (12–14). Then, in ovarian cancers and head and neck cancers, RUNX3 showed oncogenic activity (17, 19). Consistent with the human information from TCGA database, our results showed that *Runx3* knockdown in MLL-AF9-induced AML cells retarded AML progression. This indicates that RUNX3 probably plays a pro-tumor role in AML. RUNX3 regulates transcription by binding enhancers and promoters (11). So, we performed ChIP-seq in both murine normal bone marrow cells and AML cells to investigate the mechanism of the oncogenic activity of RUNX3 in AML. We further discovered that compared to that in normal bone marrow cells, RUNX3 in AML cells tended to bind genes enriched in DNA repair (*Chek1*, *Ddb1*, *Rad51c*, and *Rpa2*), antiapoptosis (*Bcl2*, *Bcl2l10*, *Bcl2l12*, and *Mcl1*), and leukemogenesis (*Myc*, *Cd93*, *Kit*, *Ikzf2*, *Fto*, and *Sox4*) pathways. *Myc* is a classical oncogene in various cancers. The activation of *Myc* by RUNX3 had already been demonstrated to be the main cause of the oncogenic function of RUNX3 (30, 31). Also, *Ddb1*, *Chek1*, and *Rad51c* are essential genes involved in DNA-damage repair of AML cells (32, 33). *Bcl2* and *Mcl1* are critical antiapoptosis genes and successfully used as treatment targets for AML (34). We demonstrated that in murine AML cells, RUNX3 mainly bound these classical genes at their promoter sites. With combined analysis of public datasets of H3K27ac ChIP-seq, we discovered that RUNX3 bound some key DNA-repair factors at their enhancer areas. However, we failed to find RUNX3 that bound any antiapoptotic factors at their enhancer areas. Our results further proved that *Runx3* knockdown significantly downregulated the expression of these DNA-repair and antiapoptotic genes in murine AML cells. This suggests that RUNX3 directly upregulates the expression of DNA-repair genes by controlling both their promoters and enhancers while upregulating the expression of antiapoptotic genes only by controlling their promoters. Decreased expression of DNA-repair genes resulted in increased DNA damage, which ultimately collaborated with the influence of reduced antiapoptotic factors to induce more apoptosis of AML cells *in vivo*. Also, our results showed that RUNX3 knockdown slightly disturbed the normal cell cycle of AML cells *in vivo*. Altogether, we elucidate that RUNX3 promotes AML progression not only by activating *Myc* transcription but also by directly regulating oncogene network covering DNA repair and apoptosis. Further studies are warranted to determine the detailed mechanism of how RUNX3 regulates the oncogene network.

Collectively, our study identified RUNX3 as an oncogene in AML, which conferred a new treatment target for AML therapy.

## Materials and Methods

### Animals

The C57BL/6 mice and CD45.1 mice (6–8 weeks old, weighing 18–22 g) were all raised in the specific pathogen-free (SPF)-level animal breeding facility of the Experimental Animal Center of Zhongshan Medical College, Sun Yat-sen University. All experimental procedures followed the experimental guidelines outlined in the Animal Care Principles and were approved by the Animal Care and Use Committee of Sun Yat-sen University.

### Definition of Enhancers and Super-Enhancers

We downloaded H3K27ac ChIP-seq data from a public database (NE SRR1915572, MO SRR787551, HSPC SRR2094192, AML1# SRR3503794, AML2# SRR3503797, AML2# SRR3503801, mouse GSE117443). Enhancers were stitched, and super-enhancers were identified using ROSE (https://bitbucket.org/young_computation/rose). Briefly, constituent enhancers were stitched together if they are within a certain distance and ranked by their input-subtracted signal of H3K27ac. And then, we separated super-enhancers from typical enhancers by identifying an inflection point of H3K27ac signal; the slope here was 1. We run ROSE with a stitching distance of 12,500 bp and allowed enhancers within 12,500 bp to be stitched together. In addition, we used a transcription start site (TSS) exclusion zone of 5,000 bp. Finally, Rose GeneMapper tool was used to annotate the genes within the 50-kb range of the super-enhancers.

### Survival Analysis of the Genes in The Cancer Genome Atlas Dataset

LAML data from TCGA were used to perform validation with the Gene Expression Profiling Interactive Analysis (GEPIA) database (http://gepia.cancer-pku.cn) (35). Furthermore, Kaplan–Meier curves were generated from the GEPIA database. The overall survival (OS) was estimated using the log-rank test, and p-value <0.05 was considered to denote statistically significant data.

### Patient Specimens

The AML patients’ specimens used in this study were derived from the routine clinical management in the Third Affiliated Hospital, Sun Yat-sen University. The procedure was approved by the ethics committee of the Third Affiliated Hospital, Sun Yat-sen University in accordance with the international guidelines and the ethical standards outlined in the Declaration of Helsinki. Mononuclear cells were isolated from the patient bone marrow with Ficoll-Hypaque and then processed to extract mRNA.

### Quantitative RT-PCR

Total mRNA was extracted from sorted GFP^+^ cells using MagZol™ Reagent (R4801-03, Magen) according to the manufacturer’s instructions. mRNA purity and quantity were determined with NanoDrop (Thermo Scientific) before qPCR analysis. For qRT-PCR, equal amounts of mRNA samples were reverse transcribed into cDNA using the TransScript All-in-One First-Strand cDNA Synthesis SuperMix for qPCR (One-Step gDNA Removal) Kit (AT341, Transgen). Quantitative real-time PCR was performed on Bio-Rad CFX96 Touch™ Real-Time PCR Detection system using SYBR Green I Master Mix reagent (11203ES03, YEASEN).

### Western Blotting

The same number of GFP^+^ bone marrow cells from control or *Runx3* knockdown AML mice was sorted into phosphate buffered saline (PBS) with 2% fetal bovine serum (FBS). The cells then were washed with PBS and lysed by radio immunoprecipitation assay (RIPA). Equal amounts of protein extracts were fractionated by 10% sodium dodecyl sulfate–polyacrylamide gel electrophoresis (SDS-PAGE) and transferred to a polyvinylidene fluoride (PVDF) membrane (IPVH00010, Merck Millipore). After being blocked with 5% non-fat milk in Tris-buffered saline with Tween-20 (TBST, pH 7.6) for 1 h at room temperature, the membranes were incubated with primary antibodies: anti-RUNX3/AML2 (D6E2) (mouse, 1:1,000, 9647, Cell Signaling Technology) and anti-β-actin (rabbit, 1:1,000, 4970, Cell Signaling Technology) overnight at 4°C and then incubated with secondary antibodies (rabbit, 1:10,000, W401B, Promega; mouse, 1:10,000, W402B, Promega) for 1 h at room temperature. The blots were detected by X-ray film or digital imaging system (Odyssey Fc).

### Acute Myeloid Leukemia Mouse Model

The 293T cells were transfected with retroviral plasmids MSCV-MLL-AF9-IRES-GFP containing MLL-AF9 and GFP cDNA sequences. Bone marrow cells from C57 mice treated with 5-fluorouracil (5-FU) for 5 days were infected with retrovirus twice with 24-h interval. The 400K infected cells were mixed with 100K protective cells to intravenously inject into WT recipient mice irradiated with a 9-Gy lethal dose. The number of animals used per experiment is shown in the figure legends.

### Constructs

*Runx3* knockdown shRNA (GAAGAGTTTCACGCTCACAAT) was cloned into pLKO.1-puro (8453, Addgene). *Runx3* knockdown and control lentivirus were prepared by HEK293T transfected by pLKO.1-puro together with psPAX2, pMD2G packaging vectors. MLL-AF9-GFP^+^ bone marrow cells were harvested from AML mice at 35 days after transplantation. These cells were infected with *Runx3* knockdown or control lentivirus and further selected by 1 μg ml^-1^ puromycin for 72 h. The 200K GFP^+^ cells screened by puromycin were mixed with 100K protective cells to intravenously inject into CD45.2^+^ recipient mice irradiated with a 4.5-Gy sublethal dose.

### γ-H2AX Immunofluorescence Staining

The cells were transferred to a glass slide and allowed to stand for 1 h to make the cells adhere to the glass slide. After fixation with 4% paraformaldehyde (PFA) for 15 min, cells were permeabilized with 0.5% Triton X-100 at room temperature for 30 min, blocked with 10% goat serum solution at room temperature for 1 h, washed, and incubated with γ-H2AX primary antibody (Biolegend, Cat 613404) overnight. After that, the secondary antibody was added dropwise and incubated at room temperature for 1 h, and the high-speed confocal imaging system (Dragonfly CR-DFLY-202 2540) was used for imaging. The γ-H2AX foci in 30 cells were counted in each group.

### Flow Cytometry

Take 20–30 μl of peripheral blood through the tail vein of the mouse and add to the anticoagulation tube. Take the bone marrow cells from the femur and tibia of the sacrificed mice. The red blood cells were lysed, and the bone marrow cells were filtered using a 100-μm cell strainer. Monoclonal antibodies to Mac-1 (M1/70, Biolegend), Gr-1 (RB6-8C5, Biolegend), c-Kit (2B8, Biolegend), Lin mix (Gr1, CD4, CD3, CD8a, Ter119, B220, IgM) (Biolegend), CD34 (MEC14.7, Biolegend), Sca1 (D7, Biolegend), FcγRII/III (93, Biolegend), IL-7Ra (A7R34, Biolegend) (all used as 50 ng per million cells) were used where indicated. After incubation with antibodies, the samples were analyzed using the Attune NxT flow cytometer (Thermo), and the results were analyzed using FlowJo software. Here, 7-aminoactinomycin D (7-AAD) (A1310, Life Technologies) was used to exclude dead cells.

### Chromatin Immunoprecipitation

Bone marrow cells were harvested from MLL-AF9-induced AML mice 35 days after transplantation, and bone marrow cells from normal syngeneic mice with the same age served as controls, five mice for each group. Here, 1% formaldehyde in PBS was used to crosslink the cells for 10 min, followed by quenching with 125 mM glycine on ice. Cells were collected and flash frozen in liquid nitrogen, then stored at -80°C for use. Frozen crosslinked cells were thawed on ice and then resuspended in lysis buffer I (50 mM HEPES-KOH pH 7.5, 140 mM NaCl, 1 mM EDTA, 10% glycerol, 0.5% NP-40, 0.25% Triton X-100, protease inhibitors). After rotating for 10 min at 4°C, the cells were collected and resuspended in lysis buffer II (10 mM Tris-HCl pH 8.0, 200 mM NaCl, 1 mM EDTA, 0.5 mM EGTA, protease inhibitors). After rotating for 10 min at 4°C, the cells were collected and resuspended in sonication buffer (20 mM Tris-HCl pH 8.0, 150 mM NaCl, 2 mM EDTA pH 8.0, 0.1% SDS, 1% Triton X-100, protease inhibitors) for sonication. Sonicated lysates were cleared once by centrifugation at 16,000 g for 10 min at 4°C. Input material was reserved as control. The remainder was incubated with magnetic beads bound with anti-RUNX3/AML2 (D6E2) antibody (mouse, 1:1,000, 9647, Cell Signaling Technology) to enrich for DNA fragments overnight at 4°C. Beads were washed with wash buffer (50 mM HEPES-KOH pH 7.5, 500 mM LiCl, 1 mM EDTA pH 8.0, 0.7% Na-deoxycholate, 1% NP-40) and TE buffer (10 mM Tris-HCl pH 8.0, 1 mM EDTA, 50 mM NaCl) in order. Beads were removed by incubation at 65°C for 30 min in elution buffer (50 mM Tris-HCl pH 8.0, 10 mM EDTA, 1% SDS). Crosslinks were reversed overnight at 65°C. To purify eluted DNA, 200 ml TE was added, and then RNA was degraded by incubation in 8 μl 10 mg/ml RNase A at 37°C for 2 h. Protein was degraded by addition of 4 μl 20 mg/ml^-1^ proteinase K and incubation at 55°C for 2 h. Phenol:chloroform:isoamyl alcohol extraction was performed followed by an ethanol precipitation. The DNA was then resuspended in 5 0ml TE. Library preparation was performed with a DNA Library Prep Kit (Vazyme, #TD501); libraries were amplified for seven cycles and were size-selected with Beckman AMPure XP beads. Two biological replicates were performed for each group.

### ChIP-Seq Data Analysis

We aligned the ChIP-Seq data to the mm9 reference genome by bowtie2 with default parameter, followed by removing the multiple aligned reads, PCR duplications with samtools. To eliminate the impact of “Problematic genomic regions”, we downloaded the ENCODE blacklist (Consortium, 2012) and discarded the reads aligning this region through bedtools. Finally, we used macs2 to calling peaks with control, setting a q value cutoff of 0.05.

### Gene Ontology Analysis

To find the GO terms enriched in RUNX3-bound genes, The clusterProfiler (36) package in R was utilized for the identification and visualization of enriched pathways among differentially expressed genes identified as described above. The functions “enrichGO” were used to identify overrepresented pathways based on the GO databases. Significance in the enrichment analysis was based on p.adjust <0.05. For **Figures 4E, G**, we reported 10 significant GO Biological Process terms and their associated q values.

### Statistics

Data are expressed as means ± SEM. For all experiments, except the determination of survival, data were analyzed by Student’s *t*-tests, and differences were considered statistically significant if p < 0.05. The survival of the two groups was analyzed using a log-rank test, and differences were considered statistically significant if p < 0.05. * p < 0.05, ** p < 0.01, *** p < 0.001.

## Data Availability Statement

The datasets presented in this study can be found in online repositories. The names of the repository/repositories and accession number(s) can be found below: NCBI BioProject PRJNA741044.

## Ethics Statement

The studies involving human participants were reviewed and approved by the ethics committee of the Third Affiliated Hospital, Sun Yat-sen University (SYSU). The patients/participants provided their written informed consent to participate in this study.

## Author Contributions

WZ, QM, BL and ZS designed and performed most of the experiments and analyzed the data. BL and LL contributed to animal experiments and patient sample assay.WZ and MZ wrote the paper. LL, DL, and MZ supervised the project. All authors contributed to the article and approved the submitted version.

## Funding

We would like to thank the National Natural Science Foundation of China (NSFC 81870127), National Natural Science Foundation of China (NSFC 81700149), Sanming Project of Medicine in Shenzhen (SZSM201911004), and Research support foundation for post doctors in the seventh affiliated hospital, Sun Yat-sen University (ZSQYRSFPD0017) for generous support.

## Conflict of Interest

The authors declare that the research was conducted in the absence of any commercial or financial relationships that could be construed as a potential conflict of interest.

## Publisher’s Note

All claims expressed in this article are solely those of the authors and do not necessarily represent those of their affiliated organizations, or those of the publisher, the editors and the reviewers. Any product that may be evaluated in this article, or claim that may be made by its manufacturer, is not guaranteed or endorsed by the publisher.

## Supplementary Material

Supplementary Figure 1
*RUNX3* is super-enhancer-associated gene in AML cells. Enhancers in three normal blood cells and three AML cells ranked based on H3K27ac signal intensity.

Supplementary Figure 2
*Runx3* knock-down is on the target. The relative mRNA expression level of *Runx1* and *Runx2* in sorted scramble control (Vector) and *Runx3* knock-down (*Runx3* KD) AML cells. Data represent mean ± s.e.m of 6 mice. ns, not significant.

Supplementary Figure 3
*Runx3* knock-down impedes cell cycle progression in AML cells. Representative FACS plots (left) and quantitative analysis (right) of cell cycle in scramble control (Vector) and Runx3 knock-down (Runx3 KD) AML cells at day 45 post-transplantation (n=5 mice).

Supplementary Figure 4 RUNX3 binds to cell cycle related genes and AML-related oncogenes both in normal bone marrow cells and AML cells. **(A, B)** Cell surface marker analysis of primary AML cells for ChIP-seq. Flow analysis of the bone marrow cells from AML mice 35 days after primary transplantation. The most commonly markers were used, such as GFP^+^CD11b^+^Gr-1^+^, GFP^+^c-Kit^+^, Gr-1^-^c-Kit^+^ **(A)**, and GFP^+^Lin^-^Sca-1^-^IL-7R^-^c-Kit^+^CD34^+^FcγRII/III^high^ **(B)** leukemia stem cells. **(C)** GO term enrichment analysis of 4667 genes that can be bound by RUNX3 in both normal bone marrow cells and AML cells. **(D)** Genome browser views of cell cycle related genes (*Mki67, Cdkn1b, Cdk4, and Ccnd1*) loci showing the distribution of RUNX3 ChIP-seq peaks. **(E)** RUNX3 directly binds to AML-related oncogenes. Genome browser views of AML-related oncogenes (*Myc, Cd93, Kit, Ikzf2, Fto* and *Sox4*) loci showing the distribution of RUNX3 ChIP-seq peaks. **(F)** Genome browser views of the distribution of RUNX3 and H3K27ac ChIP-seq peaks in DNA repair (*Rad54b, Rad51c, Chek1* and *Ddb2*) and anti-apoptosis *(Bcl-2 and Mcl-1)* related gene loci.

Supplementary Figure 5
*Runx3* knock-down inhibits the expression levels of cell cycle-related genes in AML cells. The relative mRNA expression level of cell cycle related genes which RUNX3 binds to in sorted scramble control (Vector) and *Runx3* knock-down (*Runx3* KD) AML cells. Data represent mean ± s.e.m of 6 mice. *p < 0.05, **p < 0.01, ***p < 0.001.
