# Supplementary figures and images for: Runt-Related Transcription Factor 3 Promotes Acute Myeloid Leukemia Progression
Source: Front Oncol. 2021 Oct 12;11:725336. doi: 10.3389/fonc.2021.725336 (PMC8549545; doi:10.3389/fonc.2021.725336)

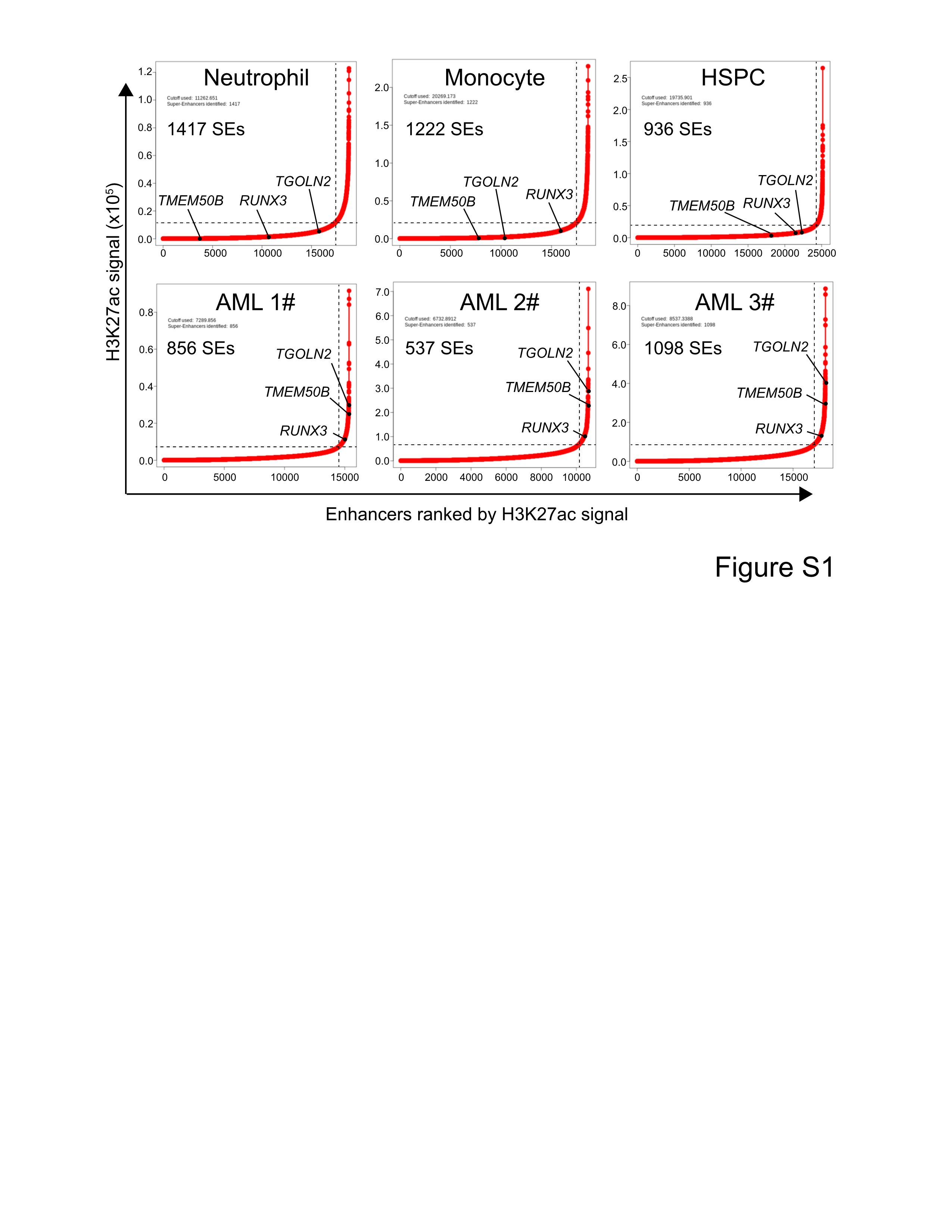

Supplement: Supplementary Figure 1 — RUNX3 is super-enhancer-associated gene in AML cells. Enhancers in three normal blood cells and three AML cells ranked based on H3K27ac signal intensity. [file Image_1.jpg]

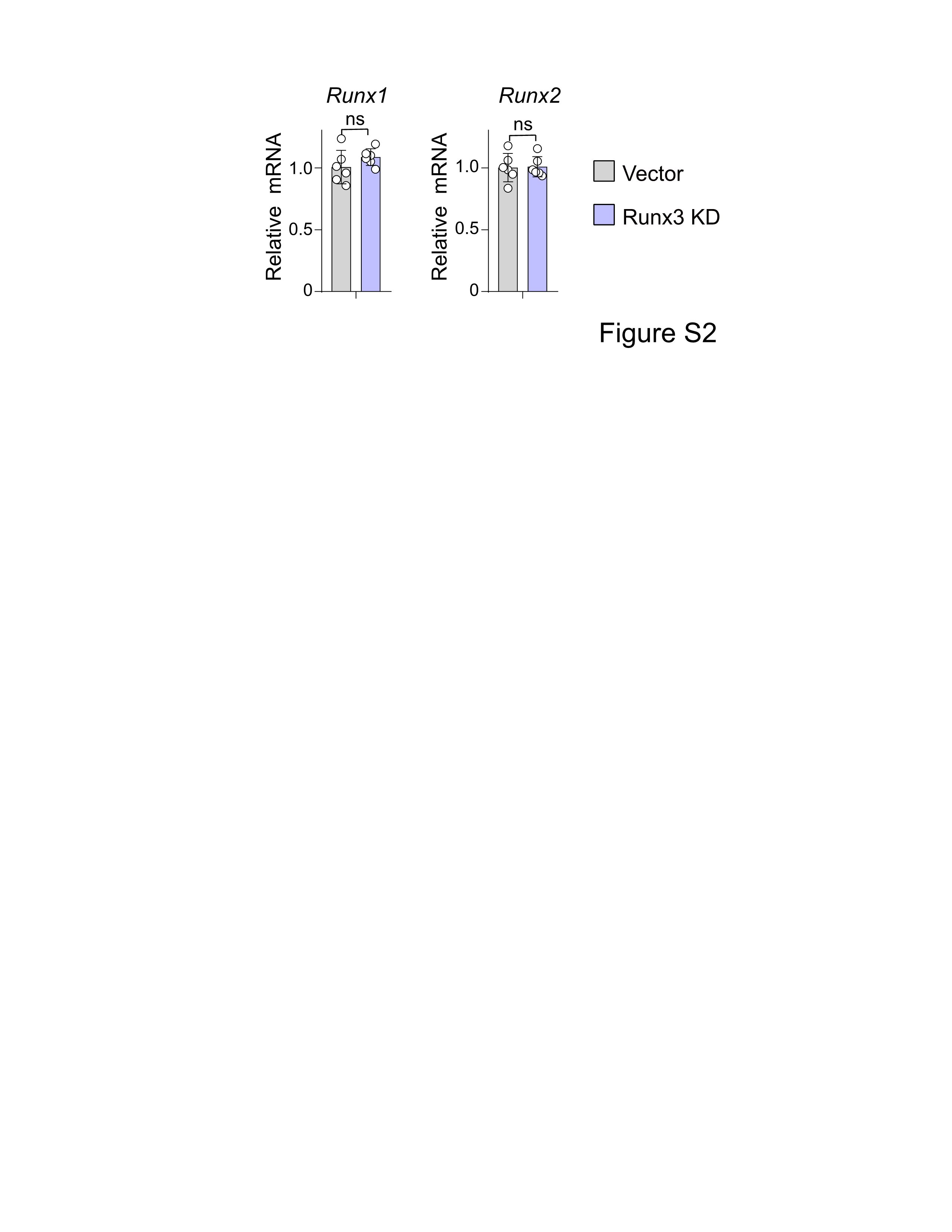

Supplement: Supplementary Figure 2 — Runx3 knock-down is on the target. The relative mRNA expression level of Runx1 and Runx2 in sorted scramble control (Vector) and Runx3 knock-down (Runx3 KD) AML cells. Data represent mean ± s.e.m of 6 mice. ns, not significant. [file Image_2.jpg]

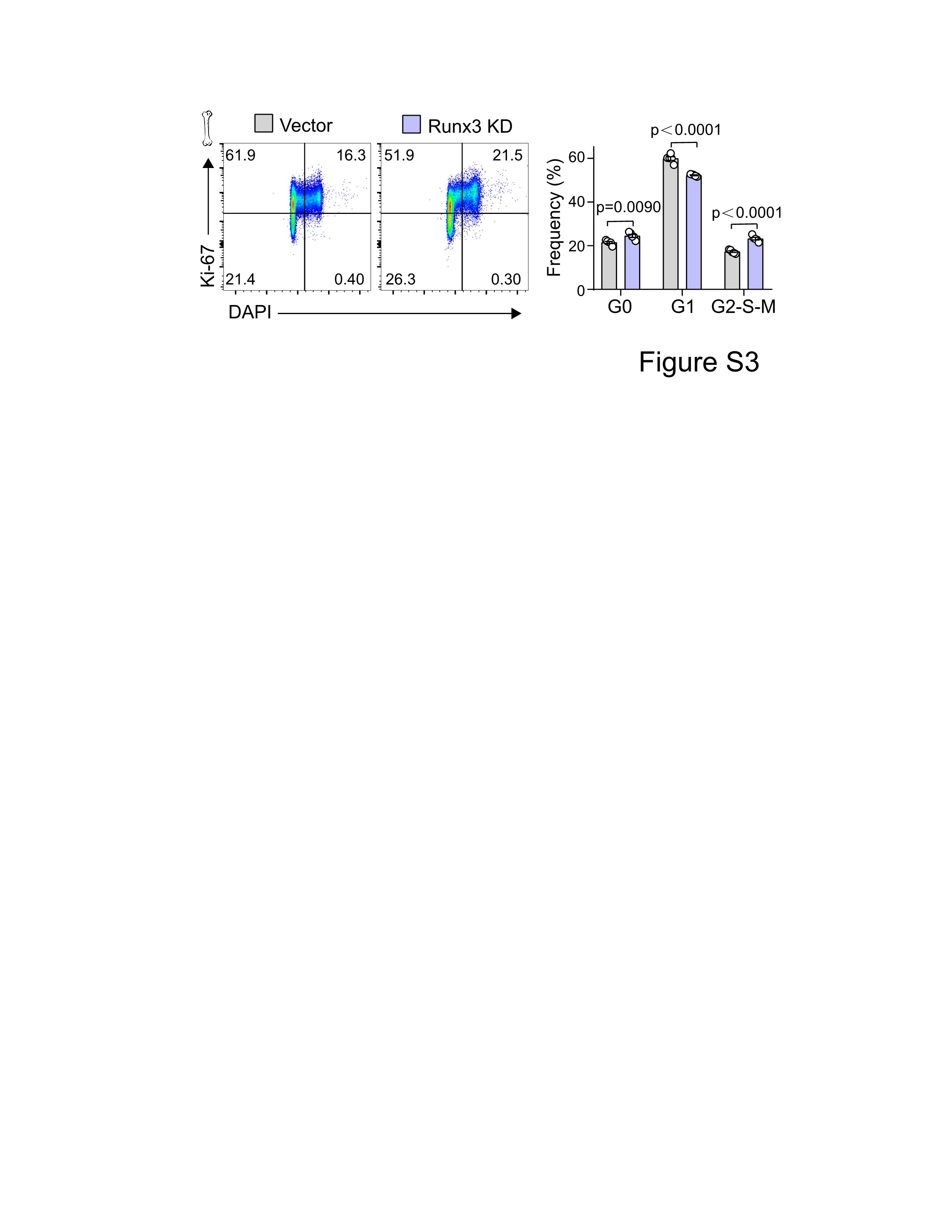

Supplement: Supplementary Figure 3 — Runx3 knock-down impedes cell cycle progression in AML cells. Representative FACS plots (left) and quantitative analysis (right) of cell cycle in scramble control (Vector) and Runx3 knock-down (Runx3 KD) AML cells at day 45 post-transplantation (n=5 mice). [file Image_3.jpg]

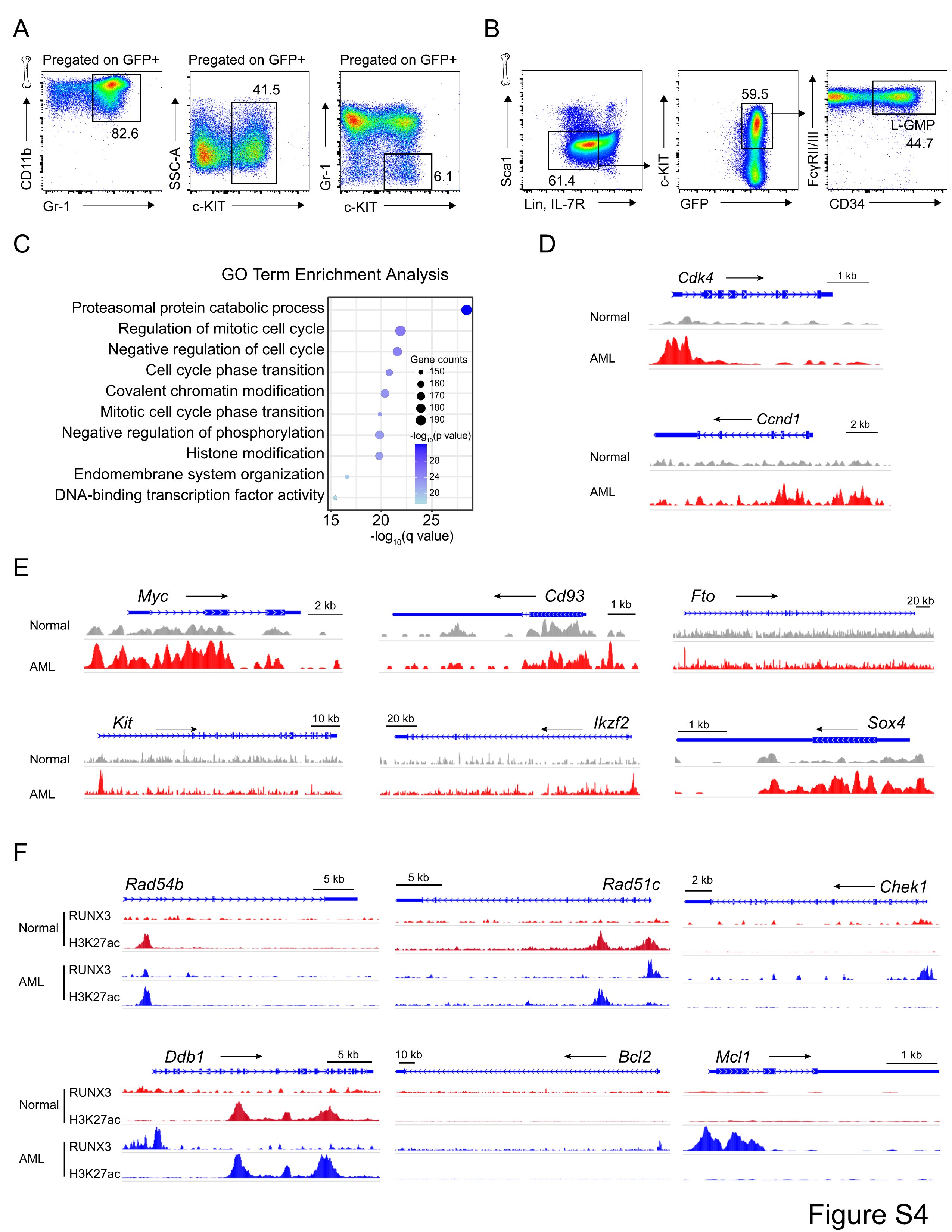

Supplement: Supplementary Figure 4 — RUNX3 binds to cell cycle related genes and AML-related oncogenes both in normal bone marrow cells and AML cells. (A, B) Cell surface marker analysis of primary AML cells for ChIP-seq. Flow analysis of the bone marrow cells from AML mice 35 days after primary transplantation. The most commonly markers were used, such as GFP+CD11b+Gr-1+, GFP+c-Kit+, Gr-1-c-Kit+ (A), and GFP+Lin-Sca-1-IL-7R-c-Kit+CD34+FcγRII/IIIhigh (B) leukemia stem cells. (C) GO term enrichment analysis of 4667 genes that can be bound by RUNX3 in both normal bone marrow cells and AML cells. (D) Genome browser views of cell cycle related genes (Mki67, Cdkn1b, Cdk4, and Ccnd1) loci showing the distribution of RUNX3 ChIP-seq peaks. (E) RUNX3 directly binds to AML-related oncogenes. Genome browser views of AML-related oncogenes (Myc, Cd93, Kit, Ikzf2, Fto and Sox4) loci showing the distribution of RUNX3 ChIP-seq peaks. (F) Genome browser views of the distribution of RUNX3 and H3K27ac ChIP-seq peaks in DNA repair (Rad54b, Rad51c, Chek1 and Ddb2) and anti-apoptosis (Bcl-2 and Mcl-1) related gene loci. [file Image_4.jpg]

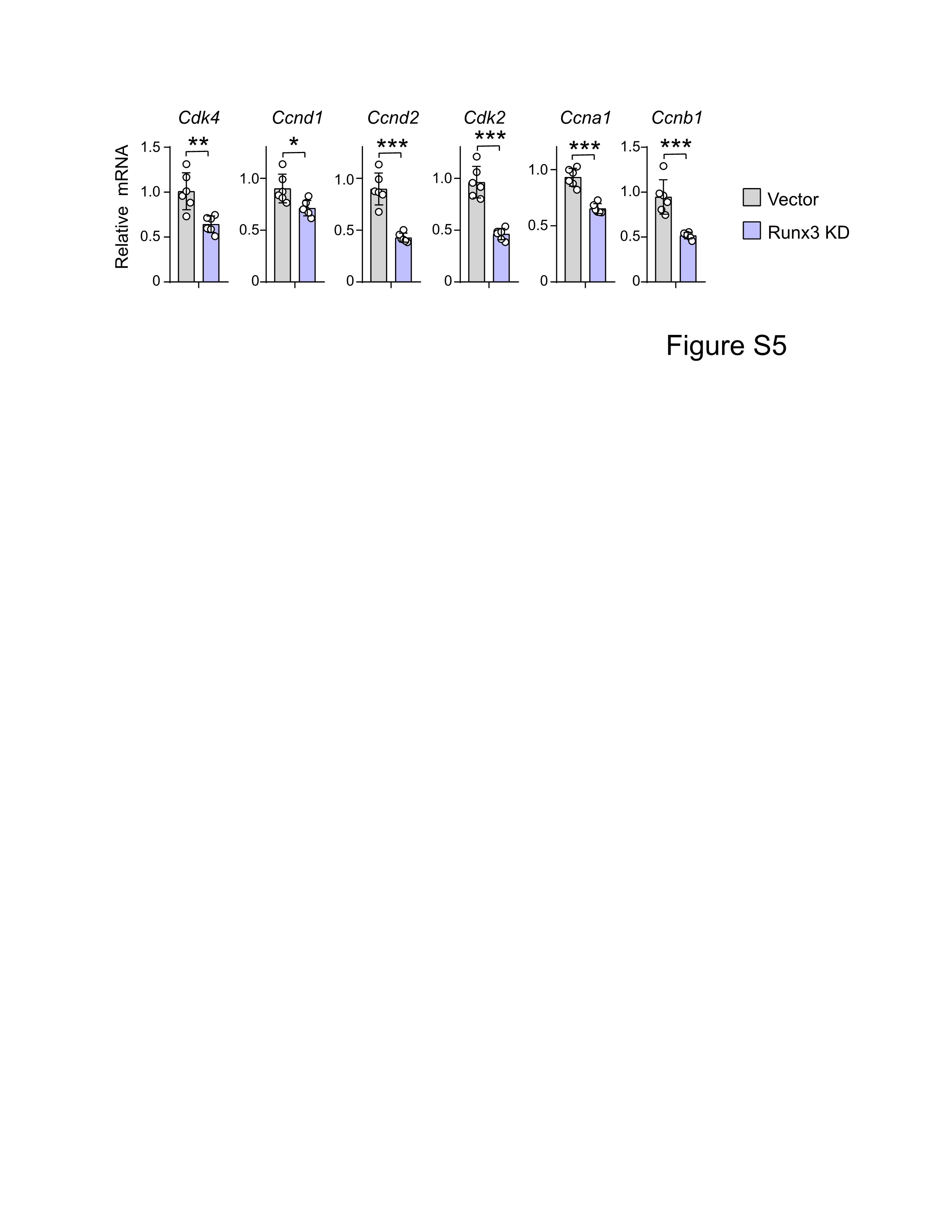

Supplement: Supplementary Figure 5 — Runx3 knock-down inhibits the expression levels of cell cycle-related genes in AML cells. The relative mRNA expression level of cell cycle related genes which RUNX3 binds to in sorted scramble control (Vector) and Runx3 knock-down (Runx3 KD) AML cells. Data represent mean ± s.e.m of 6 mice. *p < 0.05, **p < 0.01, ***p < 0.001. [file Image_5.jpg]
